# Anticytokine Autoantibodies: Association with Infection and Immune Dysregulation

**DOI:** 10.3390/antib5010003

**Published:** 2016-01-15

**Authors:** Vijaya Knight, Patricia A. Merkel, Michael D. O’Sullivan

**Affiliations:** 1 Division of Pathology, Department of Medicine, National Jewish Health, Denver, CO 80015, USA; merkelp@njhealth.org; 2 National Jewish Health Advanced Diagnostic Laboratories, National Jewish Health, Denver, CO 80015, USA; 3 Immunology Department, PathWest Laboratory Medicine WA, Perth 6009, Australia; michael.o’sullivan@health.wa.gov.au; 4 School of Pathology and Laboratory Medicine, University of Western Australia, Perth 6009, Australia

**Keywords:** cytokines, autoantibodies, interleukin, non-tuberculous mycobacteria, ARDS, chronic mucocutaneous candidiasis, SLE, rheumatoid arthritis, immune dysregulation, immune deficiency

## Abstract

The association of autoantibodies to cytokines with immune deficiency, autoimmunity and/or immune dysregulation is increasingly being recognized. For example, autoantibodies to interferon gamma have been found to be associated with chronic, treatment refractory infections with intracellular organisms such as mycobacteria, autoantibodies to interleukin 17 with chronic mucocutaneous candidiasis, and anti-interferon alpha autoantibodies with systemic lupus erythematosus. While low titer autoantibodies to these and other cytokines may be detected in normal individuals, patients with infectious or autoimmune manifestations tend to have high titer autoantibodies that may block or potentiate the function of the respective cytokine. Recognition of these autoantibodies is important because it may direct treatment toward a combination of adjunctive immunotherapy to modulate the autoantibody level while continuing with appropriate anti-microbial therapy. This review focuses on the anti-cytokine autoantibodies documented to date, their autoimmune, immune dysregulation and infectious disease associations, methods for detection of these antibodies and potential treatment options.

## 1. Introduction

Autoantibodies (AAbs) to cytokines are increasingly being recognized as potential contributors to acquired immune deficiency, immune dysregulation and autoimmunity [1,2,3]. Although definite causality has not been established in all cases, these AAbs, generally polyclonal IgG in nature, may mediate diverse infectious and/or immunological manifestations depending on the cytokine that they target. For example, AAbs to interferon gamma (IFNγ), a key mediator of protective immune responses against intracellular organisms [4], have been found to be associated with chronic, disseminated, treatment refractory infections with intracellular organisms such as mycobacteria, while AAbs to GM-CSF (Granulocyte Macrophage Colony Stimulating Factor), are associated with PAP (Pulmonary Alveolar Proteinosis), since GM-CSF orchestrates the maturation and function of pulmonary alveolar macrophages [5,6]. In other cases, such as anti-G-CSF AAbs in Felty’s syndrome, the presence of the autoantibody may be an association rather than causative of the disease [7].

The presence of anticytokine AAbs is not necessarily associated with disease; they can be detected in a majority of healthy individuals [8] and therapeutic human immunoglobulin G preparations [9]. The significance of anti-cytokine AAbs in healthy individuals has not been definitively established, and it has been suggested that they may play a role in the physiological regulation of the biological activities of cytokines either by neutralization or perhaps by prolonging their half-life by the formation of cytokine-antibody complexes [10,11]. Indeed, the natural development of thousands of IgG autoantibodies against a diverse range of human tissue antigens in healthy controls, identified by protein microarray, [12] supports speculation that they may have an important but unconfirmed physiological function. It is postulated that these tissue antigen-specific IgG AAbs may provide an adaptive mechanism to clear damaged tissue or cellular debris in certain situations such as disease or trauma. A similar hypothesis for regulatory functions of anticytokine AAbs has been proposed based on observations such as the inverse relationship between anti-interferon-alpha (IFNα) AAb titer and erosive joint disease in patients with rheumatoid arthritis (RA) [13].

The features that distinguish pathogenic from regulatory anticytokine AAbs and the factors that determine their respective development remain poorly defined. While an increasing number of publications focus on anticytokine AAbs [13], establishing their true prevalence in a range of different disease cohorts and healthy controls is confounded by the variety of techniques used to detect the AAbs in different studies, including whether free AAb and/or cytokine-antibody complexes are detected [8]. There is, however, growing evidence to suggest that anticytokine AAbs may play a direct pathogenic role in susceptibility to infection, rather than arising as a consequence of the immune response to the organism, including the detection of AAbs before development of the associated infectious disease [14] or, in the case of PAP, the capacity to reproduce disease through administration of highly purified patient-derived anti-GM-CSF AAbs to a non-human primate [15] and the ability of patient serum containing anti-IFNγ AAbs to abrogate IFNγ-mediated clearance of *Listeria monocytogenes* in peripheral blood mononuclear cell cultures [16].

While low titer anti-cytokine AAbs may be detected in healthy individuals [8], as discussed in this review anticytokine AAbs that have been associated with infectious or autoimmune complications tend to be of high titer and show *in vitro* neutralization of cytokine function. Although increasing cytokine concentration may abrogate AAb-mediated neutralization, recent *in vitro* studies of anti-GM-CSF AAbs demonstrate that the presence of multiple AAb clones can inhibit signaling regardless of cytokine concentration [17]. Appropriate recognition of these AAbs in a disease setting is important because it may direct treatment toward a combination of adjunctive immunotherapy to modulate the autoantibody titer while continuing appropriate anti-microbial therapy where necessary.

**Figure 1:**
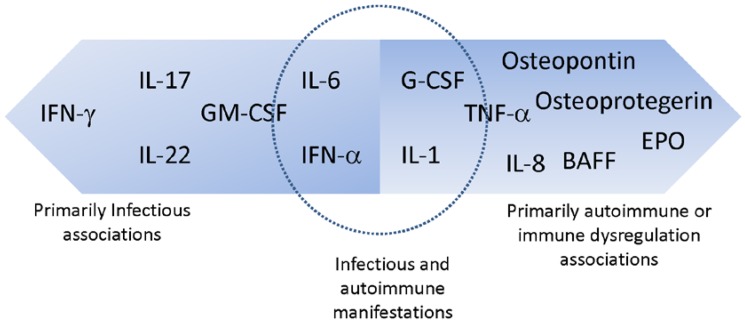
Anticytokine AAbs and disease associations.

With the advent of high throughput screening tools for the analysis of autoantibody profiles, the list of anti-cytokine AAbs detected in health and disease continues to grow [18]. Table 1 lists anti-cytokine AAbs associated with various disease states identified to date. This review focuses on the anti-cytokine AAbs for which there is growing evidence for association with infection (IFNγ, IFNα, IL-6, IL17/22, GM-CSF), with immune dysregulation/autoimmune conditions (IL-8, G-CSF, EPO) or with both (IL-6 and IFNα). As shown in Figure 1, there is considerable overlap between these categories because anti-cytokine AAbs may play a role in modulating disease activity in autoimmune conditions, as evidenced by the potentially beneficial role of anti-IFNα AAbs in modulating SLE [19], and may also increase susceptibility to infections as has been observed in certain immune deficient patients [20]. The biological significance of anti-cytokine AAbs therefore must be evaluated in the context of disease.

**Table 1:** Anti-cytokine AAbs associated with disease states identified to date.

Cytokine	Clinical associations	Possible biological role	Evidence
Interleukin-1 alpha	Non-destructive form of polyarthritis, Sjogren’s syndrome, rheumatoid arthritis, psoriasis, pemphigus.	Neutralizing, negatively correlated with disease severity, may modulate disease.	[21,22]
Interleukin-6	Recurrent staphylococcal infections, associated with low CRP levels. Observed in systemic sclerosis	Neutralizing, leads to decreased CRP levels, increased susceptibility to infection. May form stable complexes with IL-6 and contribute to disease progression in systemic sclerosis	[23,24,25,26]
Interleukin-8	Acute Respiratory Distress Syndrome	Forms immune complex with IL-8, extending proinflammatory activity and neutrophil recruitment	[27]
Interleukin-12	Autoimmune Polyendocrionopathy Syndrome type-1, thymoma associated autoimmune disease. One case of Burkholdaria lymphadenitis	Biological role not well established. Neutralizing activity may contribute to susceptibility to intracellular organisms	[28,29]
Interleukin-17/22	Autoimmune Polyendocrinopathy Syndrome type-1, Chronic Mucocutaneous Candidiasis	Neutralizing, may contribute to impaired immune responses mediated by IL-17	[30,31]
G-CSF	Felty’s syndrome, neutropenia	Not well established, may contribute to neutropenia through neutralization of G-CSF	[7]
GM-CSF	Pulmonary Alveolar Proteinosis. Intracellular infections with Cryptococcus, Norcardia, Aspergillus and Mycobacterium avium	Neutralizing, impaired alveolar macrophage development, impaired macrophage function leading to compromised cellular immune responses.	[3,32,33,34,35]
Interferon gamma	Disseminated mycobacterial infections, Infections with Salmonella typhi, CMV and Toxoplasma, reactivation of VZV	Neutralizing, abrogates IFNγ mediated cellular immune responses essential for clearance of intracellular infections	[16,36,37,38]
Interferon-alpha	Systemic Lupus Erythematosus, Autoimmune Polyendocrionopathy Syndrome type-1, Thymoma Immune deficiency associated with hypomorphic RAG mutations	Neutralizing, associated with reduction in disease severity in SLE. Neutralizing activity associated with viral infections.	[19,20,39,40]
B cell activating factor	Systemic Lupus Erythematosus	Unclear, associated with elevated levels of IFNγ and increased disease activity.	[41]
Osteopontin	Rheumatoid arthritis, prostate cancer, hepatocellular carcinoma	Unclear, may have a role in modulating disease activity in RA Potential early serum biomarker for prostate cancer. Diagnostic and prognostic biomarker for hepatocellular carcinoma	[42,43]
TNF-alpha	Systemic Lupus Erythematosus, Multiple Sclerosis	May play a role in disease modulation in SLE. Unclear role in MS.	[44,45]
Osteoprotegerin	Osteoporosis, Celiac Disease, Increased bone resorption in rheumatoid arthritis	Biological role unclear.	[46,47,48]

## 2. Anti-Cytokine AAbs Associated Primarily with Infectious Manifestations

### 2.1. Interferon Gamma (IFNγ)

Interferon gamma is one of the key cytokines involved in host defense against intracellular pathogens such as mycobacteria [4,49,50]. IFNγ, a type II interferon, is secreted chiefly by T (CD4 and CD8) lymphocytes and natural killer (NK) cells, although there is now evidence that it is produced by other cell types such as B lymphocytes, antigen presenting cells and natural killer T (NKT) lymphocytes as well [51,52,53]. IFNγ binds to its cognate receptor, a heterotetramer composed of two ligand binding chains, IFNGR1, and two signal transducing domains (IFNGR2). Binding of IFNγ to the IFNγ receptor leads to activation of Janus kinases (Jak 1 and Jak 2) followed by phosphorylation and dimerization of Signal Transducer and Activator of Transcription 1 (STAT1). STAT1 then translocates to the nucleus where it initiates transcription of IFNγ regulated genes, including MHC class II that is essential for antigen presentation, TNF-alpha and interleukin 12, both of which are essential for macrophage activation and differentiation [50]. The key role of IFNγ in generation of protective immunity to mycobacterial infections and other intracellular infections is highlighted by the fact that genetically inherited disorders of the IFNγ pathway, including IFNGR1, IFNGR2 and STAT1 (reviewed in [54]) lead to overwhelming infections with intracellular organisms of low pathogenicity such as the *Bacille Calmette Guerin* (BCG) vaccine, or non-tuberculous mycobacterial (NTM) species. These infections manifest early in childhood [54]. In adults, however, such infections are rare and are generally associated with an immune deficient state, such as HIV infection or immunosuppression following transplant [55,56].

In 2004, Doffinger *et al.* described an adult Filipino patient with AAbs to IFNγ and associated extra-pulmonary NTM infection. The patient had high titer, neutralizing antibodies to IFNγ, and succumbed to overwhelming infection [57]. Over the subsequent 10 years, several reports have documented the association of AAbs to IFNγ with intracellular infections in otherwise healthy, immune competent individuals [16,58,59]. In general, extra-pulmonary, disseminated, NTM infections were reported in these patients, although infections with *Salmonella typhi*, cytomegalovirus, cerebral toxoplasmosis and reactivation of varicella zoster [37] have been reported as well. While disseminated extra-pulmonary infections in otherwise healthy individuals are commonly observed, DeLeon *et al.* noted the presence of anti-IFNγ AAbs in a patient with isolated NTM empyema [36].

The common features of this autoimmune phenomenon that may contribute to an immune deficient state, are that patients are, in general, otherwise healthy, not obviously immune compromised, and are to a large extent of Southeast Asian origin, although very recently two cases of anti- IFNγ AAb associated infections have been reported in non-Asian patients [37,60]. In general, these patients tend to manifest with extra-pulmonary infections with NTM, and pulmonary infections are rare. IFNγ AAbs in these patients are neutralizing in nature and of very high titer.

The predominance of AAbs to IFNγ in patients of Southeast Asian origin is strongly suggestive of an inherited predisposition to development of these AAbs. Indeed, these AAbs have now been shown to be associated with HLA types HLA-DRB1*16:02 and HLA-DQB1*05-02 [58] suggesting that the development of these antibodies may be similar to those observed in other HLA-linked autoimmune diseases, such as celiac disease. However, the recent identification of patients of non-Asian origin suggest that this autoimmune phenomenon may be more wide-spread than previously thought and is perhaps influenced by other, as yet unidentified, genetic and/or environmental factors.

AAbs to IFNγ have been demonstrated by the ability of patient’s plasma to inhibit phosphorylation of STAT1 in normal monocytes stimulated with IFNγ [61], as well as by binding studies to evaluate titer. It is essential to demonstrate the functionality of these antibodies, since healthy individuals may show evidence of low titer anti- IFNγ AAbs with little to no neutralizing activity *in vitro*. Additionally, at least one patient has been identified by an initial indeterminate Quantiferon Gold In-tube result since the high titer, neutralizing anti- IFNγ AAbs in plasma interfere with the detection of IFNγ in mitogen-stimulated whole blood (unpublished observation).

### 2.2. Interleukin-17 (IL-17) and Interleukin-22 (IL-22)

There are six members of the interleukin-17 cytokine family, namely IL-17 A through F [62]. IL-17A and IL-17F are the most intensively studied due to their role in both protective immunity against *Candida albicans* [63] and their capacity to promote inflammation in autoimmune diseases such as rheumatoid arthritis and psoriasis [64,65]. IL-17A and IL-17F are produced by the Th17 subset of T cells, and form homo- or heterodimers to act on epithelial cells to induce antimicrobial peptide expression and promote neutrophil trafficking [63]. IL-22, a member of the IL-10 cytokine family, is produced by a variety of innate and adaptive immune cells and promotes epithelial barrier function, antimicrobial peptide synthesis, and production of pro-inflammatory cytokines to aid in host defense [66].

Chronic mucocutaneous candidiasis (CMC) is a clinical syndrome characterized by recurrent or persistent superficial skin, nail and mucosal infection with *Candida* organisms, usually *C. albicans* [67]. A diverse range of conditions predispose patients to mucocutaneous candidiasis. Patients with profoundly impaired cellular immunity, such as those with a primary T cell immunodeficiency or acquired immunodeficiency secondary to HIV infection, are at risk of severe oropharyngeal candidiasis as well as other opportunistic infections, and mucosal candidiasis may also be seen a side effect of antibiotics or immunosuppressants, including corticosteroids [67,68].

The central role of IL-17 cytokines in mucocutaneous immunity to *Candida* has been supported by descriptions of the autosomal dominant hyper IgE syndrome [69], CARD9 deficiency [70] and dectin-1 deficiency [71], all of which are associated with impaired Th17 cell development and susceptibility to mucocutaneous candidiasis, as well as mutations in the genes encoding IL-17 receptor A (IL-17RA) and IL-17F in patients with CMC [72].

The combination of autoimmune endocrinopathy and CMC was first described in 1929 [73] and subsequently the autoimmune polyendocrinopathy syndrome type I (APS-I), also known as autoimmune polyendocrinopathy, candidiasis-ectodermal dystrophy (APECED) syndrome, was defined on the basis of organ-specific autoimmunity involving multiple endocrine glands and CMC [74]. The underlying molecular defect causing APECED was confirmed in 1997 to be a deficiency of the autoimmune regulator (AIRE) expression in the thymus due to autosomal recessive *AIRE* mutation, resulting in a breakdown of self-tolerance [75]. Defective AIRE expression was subsequently identified in tumor tissue from thymoma patients, who are at risk of developing a variety of autoimmune diseases such as myaesthenia gravis, bone marrow aplasias and systemic lupus erythematosus [76] but have a different spectrum of clinical manifestations than patients with APECED.

While understanding the central role of *AIRE* as an autoimmune regulator gene provided an explanation for the polyendocrinopathy in APECED, the mechanism underlying susceptibility to CMC remained uncertain. The mechanistic link between autoimmunity and CMC was established in 2010 with the publication of two papers describing AAbs to IL-17A, IL-17F and IL-22 in patients with APECED [30,31]. Puel and colleagues detected high titer AAbs to one or more of IL-17A, IL-17F and/or IL-22 in 33 patients with APECED, but not in patients with other autoimmune disease or healthy controls, and demonstrated functional inhibition of IL-17A *in vitro* using plasma from one of these patients [31]. Kisand and colleagues reported on a cohort of 162 patients with APECED and described anti-IL-17A, IL-17F and/or IL-22 AAbs in over 90% of cases, and additionally detected high titer IL-17A and IL-22 AAbs in two patients with thymoma and CMC [30]. The presence of anti-IL-17 and/or IL-22 AAbs does not provide a complete explanation for susceptibility to CMC in these cohorts; some patients with CMC did not have detectable AAbs and conversely others had AAbs but not CMC. This phenomenon was also described by Wolff and colleagues in a separate cohort of 16 children with APECED [14], suggesting the existence of other susceptibility factors in these patients that are yet to be defined.

### 2.3. Granulocyte-Macrophage Colony Stimulating Factor (GM-CSF)

Granulocyte-macrophage colony-stimulating factor (GM-CSF) promotes the proliferation and differentiation of hematopoietic cells including macrophage precursors, neutrophils and dendritic cells. Initially described in mice and subsequently identified in humans, GM-CSF induces the terminal differentiation of alveolar macrophages through the transcription factor PU.1 [5] and also primes the antimicrobial functions of neutrophils [77].

The critical role of GM-CSF in the terminal differentiation of alveolar macrophages was highlighted in GM-CSF deficient (GM−/−) mice [78,79]. These mice had no detectable abnormalities of peripheral blood cells or bone marrow progenitors, but developed widespread accumulation of an amorphous, acellular material in the alveolar spaces of the lungs. This material was confirmed to be pulmonary surfactant, and the lung disease present in the GM−/− mice was noted to resemble that of the disease pulmonary alveolar proteinosis (PAP) seen in humans [78,79]. The accumulation of these surfactant proteins in GM−/− mice was due to impaired catabolism by alveolar macrophages, and normal function could be restored with chronic administration of exogenous GM-CSF [80].

Pulmonary alveolar proteinosis (PAP) is a rare disease in humans and may be congenital, secondary or acquired [81]. Following its first description in 1958 [82], the acquired form of the disease was termed idiopathic PAP until the identification of neutralizing factors for GM-CSF in the bronchoalveolar lavage (BAL) fluid of patients with PAP [83]. In these patients, GM-CSF levels in plasma and BAL fluid were found to be elevated, and isolated alveolar macrophages responded normally to stimulation with GM-CSF, providing evidence against GM-CSF deficiency or impaired signaling in alveolar macrophages as the pathogenic mechanism [84]. Kitamura and colleagues confirmed the binding factor for GM-CSF in BAL fluid of these patients to be an IgG antibody with capacity to selectively neutralize the growth of a GM-CSF-dependent cell line, and also identified the presence of the antibody in the patients’ serum [83]. A subsequent study of 40 patients with a confirmed diagnosis of acquired PAP detected anti-GM-CSF antibodies in the serum and BAL fluid of all patients, using ELISA plates coated with recombinant GM-CSF as the target antigen [85].

Prior to the identification of neutralizing anti-GM-CSF AAbs, impairment of alveolar macrophage adhesion, chemotaxis, phagocytosis and killing had been described in patients with PAP [86,87]. More recently, impairment of neutrophil phagocytosis, adhesion, oxidative burst and bactericidal activity were demonstrated in patients with PAP and shown to be reproducible in normal control neutrophils in the presence of anti-GM-CSF AAbs from patient serum [35].

These observations were thought to provide some explanation for the over-representation of opportunistic pathogens in patients with acquired PAP, including *Mycobacterium avium* complex, and *Cryptococcus, Norcardia* and *Aspergillus* species [88]. Some of these patients developed opportunistic extrapulmonary (particularly central nervous system) infections, and informed the basis for review of 107 serum and CSF samples from patients with cryptococcal meningitis from which anti-GM-CSF antibodies were identified in seven cases [33], of whom two subsequently developed features of PAP. AAbs from these patients were shown to inhibit GM-CSF-induced STAT5 phosphorylation in a flow cytometric assay of peripheral blood mononuclear cells [32]. Anti-GM-CSF AAbs have also been described in five otherwise immunocompetent patients with disseminated *Nocardia* infection [33], and these recent descriptions have expanded the spectrum of clinical presentations in which the presence of anti-GM-CSF antibodies should be sought.

The basis for the diverse clinical phenotypes associated with anti-GM-CSF antibodies remains unclear. Therapeutic trials of a monoclonal anti-GM-CSF antibody in rheumatoid arthritis have not reported any cases of PAP as a complication of therapy [89]. Some explanation for this observation may be provided by Piccoli and colleagues from their studies of the neutralizing capacity of monoclonal anti-GM-CSF antibodies [17]. They demonstrated that the neutralizing effect of monoclonal antibodies was diminished by increasing the GM-CSF concentration in a bioassay, but that a combination of three non-cross-competing monoclonal antibodies could completely neutralize GM-CSF, regardless of assay conditions, in a similar fashion to polyclonal anti-GM-CSF AAbs from PAP patients. Their results suggested that polyclonal anti-GM-CSF AAbs lead to inhibition of GM-CSF through irreversible sequestration in high molecular weight immune complexes and Fc-dependent degradation, whereas therapeutic monoclonal antibody-bound GM-CSF accumulates *in vivo* and could dissociate and bind to its receptor, potentially preserving sufficient GM-CSF functional activity to prevent the development of PAP and susceptibility to opportunistic infection. However, detectable anti-GM-CSF AAbs have predated the development of clinical disease in some patients [33], so ongoing vigilance will be required in monitoring for immune and infectious complications of therapeutic monoclonal anti-GM-CSF.

## 3. Anti-Cytokine AAbs Associated with both Infectious and Autoimmune Manifestations

### 3.1. Interleukin-6 (IL-6)

Interleukin-6 is a pleiotropic cytokine that plays a major role in inflammation and acute-phase responses, as well as in adaptive immune responses [90]. It is produced by a variety of cells, including macrophages, lymphocytes and hepatocytes. IL-6 is a regulator of the acute phase response in the liver, inducing the production of C-reactive protein (CRP), serum amyloid, fibrinogen and other acute phase mediators [90].

Binding of IL-6 to its cognate receptor leads to association of the IL-6 receptor with gp130, the signaling domain for this ternary complex. Upon binding of IL-6 to the IL-6 receptor, STAT3 is phosphorylated and translocates to the nucleus, where it binds to the promoters of IL-6 responsive genes [90]. The importance of this pathway in mediation of protective responses against bacterial infections is evident from the fact that patients with Hyper IgE Syndrome (HIES), caused by mutations in STAT3, are susceptible to recurrent staphylococcal infections and abscesses [91].

Naturally occurring AAbs to IL-6 were first described in patients with cirrhosis of the liver. In this cohort of patients, the presence of anti-IL-6 AAbs were significantly associated with increased frequency of infections [92]. Suzuki, Takemura *et al.* noted that anti-IL-6 AAbs were increased in serum of patients with systemic sclerosis, concomitantly with elevated levels of IL-6. They proposed that anti-IL-6 AAbs acted as a carrier, stabilizing IL-6 in circulation and enabling delivery of the cytokine to target cells and tissues [24,25]. Given the role of IL-6 in inflammatory autoimmune conditions, it is conceivable that these immune complexes could contribute to disease progression [93]. Puel *et al.* noted the association of neutralizing anti-IL-6 antibodies and undetectable CRP levels in a Haitian child with recurrent staphylococcal cellulitis and subcutaneous abscesses [23]. Additionally, Nanki *et al.* described two patients with severe bacterial infections that also showed evidence of anti-IL-6 antibodies in circulation, along with depressed C-reactive protein levels [26]. They hypothesized that the neutralizing activity of anti-IL-6 autoantibodies may have resulted in a block of IL-6 signaling and therefore inhibiting the production of CRP, thus leading to severe bacterial sepsis.

Excessive IL-6 production has been implicated in the development of systemic inflammatory response syndrome (SIRS) and cytokine release syndrome (CRS) following infection or tissue injury. Additionally, dysregulation of IL-6 production plays a role in chronic autoimmune diseases such as rheumatoid arthritis. Blocking excessive IL-6 may be advantageous in limiting autoimmune phenomena in these patients; however, it must be balanced with the potential for neutralization of IL-6 to increase susceptibility to infection as discussed earlier. Humanized IL-6 pathway antagonists, such as tocilizumab (anti-IL-6R) have been used successfully in rheumatoid arthritis, Castleman’s disease and juvenile idiopathic arthritis [94,95,96], and may have a role in SIRS and CRS. Since the pathological processes in these diseases are mediated by elevated levels of pro-inflammatory cytokines including IL-6, neutralization of the cytokine plays a significant role in modulation of the disease. It may be important to consider the potential for infections in patients on long term therapy with IL-6 antagonizing biologicals. Yamamoto *et al.*, in a 3-year study of 5573 rheumatoid arthritis patients on Tocilizumab noted that the rate of infections was not increased in these patients [97] whereas Gerd Horneff noted an increased incidence of infections in juvenile rheumatoid arthritis patients on Tocilizumab [98]. Ongoing vigilance and observation of patients on anti-IL-6 biologics is therefore warranted.

### 3.2. Interferon-Alpha (IFNα)

Type 1 interferons were described over 50 years ago as the factor responsible for viral interference [99]. Type 1 interferons include thirteen IFNα subtypes and IFNβ, ω, ϵ and κ [100]. All type 1 IFNs share a ubiquitously expressed heterodimeric receptor composed of the IFNAR1 and IFNAR2 chains. Binding of the type 1 IFNs to their cognate receptor initiates signaling through the Janus kinases Jak1 and Tyk2, resulting in formation of STAT1-STAT2 heterodimers that translocate to the nucleus to initiate transcription of interferon-responsive genes [100].

The role of type 1 interferons in the immune response is complex. Type 1 IFNs regulate both the adaptive and innate responses and act directly or indirectly on a variety of immune cells, including NK, T, B, antigen presenting and phagocytic cells [101]. Exposure of cells to type 1 IFNs induces an anti-viral state and early production of interferons during viral infection limits viral replication [101]. In bacterial infections, however, the role of type 1 IFNs is complex and elevated levels of the cytokine may be deleterious [101]. Type 1 IFNs increase the susceptibility of lymphocytes and macrophages to apoptosis-inducing stimuli. Since these cells are key mediators of cellular immunity to intracellular organisms such as *Listeria monocytogenes* and *M. tuberculosis*, elevated levels of type 1 IFNs may be deleterious to control of such infections. Indeed, type 1 IFN-receptor deficient mice have been shown to be more resistant to infection with *Listeria monocytogenes* compared with wild-type mice [102] and the ability of *M. tuberculosis* strains to induce elevated levels of type 1 IFNs in mice has been correlated with increased virulence [103]. Type 1 interferons also play a central role in the pathogenesis of systemic lupus erythematosus (SLE). Hooks *et al.* noted that elevated levels of type 1 IFNs in the serum of patients with SLE correlated with disease activity [104,105]. Additionally, it was noted that some patients who were treated with type I IFNs for chronic infections or malignancy developed a lupus-like syndrome that was very similar to SLE [106,107]. Microarray analysis of peripheral blood mononuclear cells from SLE patients indicated overexpression of a large number of IFN-stimulated genes [108,109], confirming the role of type 1 IFNs in the pathogenesis of SLE.

Autoantibodies to type 1 IFNs, in particular to IFNα, have been noted in patients with SLE. In a study of 49 SLE patients, Morimoto and colleagues noted AAbs to IFNα in 27% of these patients. Presence of neutralizing antibodies to IFNα in these patients was associated with decreased biological activity of circulating IFNα as well as with lowered disease severity [39]. In addition, Ching *et al.*, in a survey of 76 SLE patients found that 42% demonstrated significant titers of autoantibodies to one or more of the type 1 IFNs and furthermore, that sera from patients with clinically quiescent disease demonstrated higher levels of anti- IFNα autoantibodies [19] and hypothesized that AAbs to type 1 IFNs may therefore play a role in modulating severity of disease.

High titer AAbs to type 1 IFNs have also been described in APS-1, resulting from mutations in the AIRE gene that directs the development of self-tolerance [76], and in association with thymoma that constitute an autoimmunogenic environment [40]. APS-1 is characterized by multiple autoimmune processes, involving the endocrine system in particular. Sera from APS-1 patients demonstrate autoantibodies to the IL17/IL22 family of cytokines (reviewed earlier), IL-12 and the type 1 IFNs [76]. While the increased incidence of CMC in these patients may be attributed in part to IL17/IL22 autoantibodies, AAbs to type 1 IFNs do not appear to be associated with increased risk of viral infections in these patients. AAbs to type 1 IFNs have also been recently noted in immune deficient patients with hypomorphic RAG mutations [20]. In a study of 30 RAG-deficient patients, Walter *et al.* noted that these patients, particularly those with combined immune deficiency associated with granulomatous disease and/or autoimmunity tended to produce a broad spectrum of AAbs, in particular, AAbs to type 1 IFNs. The elevated levels of AAbs to IFNα in these patients were also associated with repeated and severe viral infections, suggesting that these AAbs may be, in part, responsible for increased susceptibility to viral infections.

AAbs to type 1 IFNs, and IFNα in particular, therefore may play differing roles depending on the disease state. The association of elevated AAbs to IFNα in quiescent SLE suggests that anti-IFNα AAbs could potentially be a useful biomarker for disease progression. In contrast to the role of anti-IFNα AAb in autoimmune disease, high titers of the AAb may play a deleterious role in the setting of immune deficiency by increasing susceptibility to viral infections [20].

### 3.3. Granulocyte Colony-Stimulating Factor (G-CSF)

Granulocyte Colony-Stimulating Factor (G-CSF) is produced by bone marrow stromal cells and is central to the maturation, differentiation and survival of neutrophils [110]. It is produced in response to physiological stress, such as infection, and acts on myeloid precursors to increase the output of neutrophils from the bone marrow. The central role played by G-CSF in neutrophil development is evident from the fact that G-CSF −/− or G-CSFR −/− mice demonstrate severe, chronic neutropenia [111,112].

Autoantibodies to G-CSF have been noted in healthy individuals, although their significance is unknown. While a direct association of naturally occurring anti-G-CSF autoantibodies with infection has not been observed to date, these autoantibodies have been observed in patients with Felty’s syndrome [7], an autoimmune condition characterized by a clinical triad of rheumatoid arthritis, splenomegaly and neutropenia, and in SLE patients. In this cohort of patients, anti-G-CSF autoantibodies were found to be associated with elevated G-CSF levels, and *in vitro* studies demonstrated the ability of the autoantibodies to neutralize G-CSF, suggesting that anti-G-CSF autoantibodies may contribute, in part, to neutropenia in these individuals [7]. Most individuals with Felty’s syndrome are generally asymptomatic; however, others may develop life threatening infections secondary to neutropenia. The causality of G-CSF AAbs with neutropenia in these patients has not been firmly established.

## 4. Anti-Cytokine AAbs Associated with Immune Dysregulation or Autoimmune Manifestations

### 4.1. Interleukin 8 (IL-8)

Interleukin 8, or neutrophil activating protein (NAP-1) is a potent chemoattractant for neutrophils [113]. IL-8 belongs to the CXC chemokine family. The 99 amino acid long translational product of the IL-8 gene undergoes proteolytic cleavage to yield several differently processed biologically active products, and is thought to exist as a non-covalently linked dimer in solution [114]. The biological effects of IL-8 include attraction of neutrophils to the site of inflammation, stimulation of release of neutrophil granules, induction of rearrangement of the cytoskeleton and activation of integrins and oxidative burst, thereby influencing all aspects of neutrophil function [115,116,117,118]. Elevated levels of IL-8 are observed in a variety of inflammatory conditions.

AAbs to IL-8 have been observed in acute respiratory distress syndrome (ARDS). ARDS, characterized by severe respiratory failure, was first described in 1967 [119]. A variety of clinical causes including surgery, sepsis, pneumonia and trauma can contribute to the development of ARDS. Immune dysregulation is considered to be characteristic of ARDS. The underlying pathology is increased permeability of the pulmonary and vascular epithelium as a consequence of microvascular damage due to the initial systemic inflammatory response. Bronchoalveolar lavage fluid in ARDS shows increased levels of IL-8, often associated with anti-IL-8 AAbs [27]. Unlike many other AAbs to cytokines that may mediate pathological processes by neutralizing the beneficial action of the target cytokine, IL-8:IL-8 AAb complexes are thought to prolong the pro-inflammatory action of IL-8. Fudala *et al.* [27] showed that IL-8:IL-8 AAb complexes induced severe lung inflammation in rabbits. IL-8:IL-8 AAb complexes have been shown to inhibit neutrophil apoptosis by increasing the levels of the anti-apoptotic protein BcL-xL as well as suppressing caspase 3 and 9, and the pro-apoptotic proteins Bax and Bak [120]. In addition, immune complexes act through activating receptors CD32 and CD16, present on a variety of immune cells. Binding of immune complexes to these activating receptors elicit cellular activation, phagocytosis, endocytosis and thus prolong inflammatory responses.

Immune complexes formed from anti-HLA antibodies or anti-neutrophil antibodies and their specific antigens are implicated in transfusion related acute lung injury (TRALI) [121,122]. IL-8:IL-8 AAbs complexes may play a similar role in ARDS and contribute to the ongoing pathogenesis and severity of disease.

### 4.2. Erythropoietin (EPO)

Erythropoietin (EPO) is a cytokine that is essential for erythropoiesis. Erythropoiesis was first described in 1906 when two French scientists, Carnot and DeFlandre, reported that small amounts of plasma from an anemic rabbit injected into a healthy rabbit caused a rapid increase in red blood cell production [123,124]. EPO is produced by the liver in the developing fetus and in the interstitial fibroblasts in the kidney and liver after the neonatal period. EPO induces erythropoiesis by acting through the EPO receptor (EpoR) present on red blood cell (RBC) progenitors and precursors in the bone marrow to promote survival, proliferation, differentiation, and maturation of RBC [125]. EPO gene expression is induced by hypoxia through the transcription factor hypoxia inducible factor-1 (HIF-1) [123,124]. Signaling through the EpoR includes the Janus family tyrosine protein kinase 2 (JAK-2) and MAP kinase pathways, leading to downstream activation of ERK1/2, PI3K/AKT, NFK-B, and STAT-5 [126]. The EpoR is found on numerous tissues, and EPO is being investigated for its role in neuroprotection, especially for the treatment of ischemic brain injury [126,127,128], and plays a role in cell survival, neurogenesis, white matter protection/regeneration, as well as anti-inflammatory and pro-angiogenic processes.

Through recombinant DNA technology, recombinant EPO (rhEPO) is now one of the most widely used drugs to treat anemia in patients with chronic kidney disease (CKD), acquired immune deficiency syndrome, multiple myeloma, myelodysplastic syndromes, and chemotherapy-induced anemia. This treatment, however, has led to rare cases of the development of anti-EPO antibodies (anti-EPO AAbs). AAbs to EPO can neutralize the protein and obliterate RBC production in the bone marrow, leading to a devastating condition known as antibody-mediated pure red cell aplasia (PRCA). PRCA is characterized by an absolute resistance to EPO therapy and a sudden drop in hemoglobin concentration due to the complete cessation of RBC production. Blood reticulocyte count is very low (<10,000/mm^3^) and the hemoglobin concentration declines at a weekly rate of about 1 g/dL, requiring RBC transfusions. Bone marrow examination shows almost complete absence of erythroid precursors but normal platelet and white cell precursors. The vast majority of cases of patients developing AAbs to erythropoietin (EPO) are correlated with long-term usage of erythropoiesis-stimulating agents (ESAs) in patients with chronic kidney disease (CKD) [129,130,131]. There are as few as three reports describing patients developing the AAbs to EPO that were never treated with ESAs [132,133,134]. Antibody-mediated PRCA is rare, with an exposure adjusted incidence of 0.02–0.03 per 10,000 patient-years [129]. The antibodies are capable of neutralizing the recombinant as well as endogenous erythropoietin, and also cross react to all known biologics, thus treatment with an alternative ESA is ineffective. Postulations explaining the mechanism behind the production of AAbs to exogenous EPO include improper handling of the biologic (a break in the cold storage chain), changes in formulation, the route of administration (subcutaneous versus intravenous administration), the duration of therapeutic treatment with the ESA, and patient characteristics that might render them more prone to develop AAbs to EPO [129,131,135,136,137].

Anti-EPO antibodies may be detected by traditional binding assays such as ELISA, radioimmunoprecipitation assay (RIPA), and the biosensor immunoassay (BIAcore). These methods do not address the functionality of the antibodies and, in addition, have varying degrees of sensitivity and specificity. Functional assays for anti-EPO AAbs include demonstration of inhibition of growth of erythroid progenitors or EPO-dependent cell lines [130,131,132,136].

## 5. Treatment

The treatment of those patients in whom anti-cytokine AAbs have been demonstrated to play a role in disease pathogenesis (GM-CSF, IFNγ and EPO) is challenging, and can be broadly divided into those that focus on the clinical disease manifestations, and those that aim to eliminate or bypass the neutralizing effect of the autoantibodies.

The former group, which includes approaches such as whole lung lavage for patients with pulmonary alveolar proteinosis [88], antimicrobial drugs for the infective complications of anti-IFNγ [138], anti-IL-17 [31] or anti-GM-CSF autoantibodies [33], or RBC transfusions in the case of anti-EPO mediated PRCA [129,130] may lead to temporary clinical improvement; however, the patients may remain at risk of recurrence or treatment failure due to persistence of the anticytokine AAb.

Chronic mucocutaneous candidiasis can usually be treated with topical and/or systemic antifungal agents, so more aggressive immunomodulatory treatments are generally not required in patients with anti-IL-17 and IL-22 autoantibodies.

Exogenous GM-CSF has been administered both subcutaneously and by inhalation in patients with PAP. Studies of subcutaneous recombinant human GM-CSF (rhGM-CSF) administration in patients with PAP have reported a clinical response in 43 to 48% of patients [139,140]. The mechanism by which exogenous GM-CSF leads to clinical response is not definitively established. The observation by Seymour and colleagues that treatment responders had a higher peak eosinophil count than non-responders suggests that at least part of the treatment efficacy may be dependent on overcoming the neutralizing effect of anti-GM-CSF antibodies [139].

There are conflicting reports on the relationship between anti-GM-CSF antibody titer and treatment response with subcutaneous rhGM-CSF. One study reported a correlation between anti-GM-CSF titer and disease activity, with only responders having a decline in titer during the course of the study [140], while another found no difference in titer according to treatment response [34]. Amongst 39 Japanese patients with anti-GM-CSF autoantibodies and PAP recruited for a study evaluating inhaled rhGM-CSF, 62% responded with improved oxygenation, with no difference in antibody titer between responders and non-responders and no change in titer amongst all subjects during the course of the study [141]. The experience of immune tolerance induction in patients with hemophilia due to inhibitory factor VIII antibodies, showing that repeated administration of exogenous factor VIII can ultimately result in tolerance in a majority of patients [142], provides some support for the hypothesis of tolerance induction with rhGM-CSF in PAP.

In view of the pathogenic nature of some anticytokine AAbs, immunomodulatory treatment approaches such as plasmapheresis and rituximab, similar to those used for well-described autoantibody-mediated diseases (e.g., myaesthenia gravis [143]), have been employed. Conventional immunosuppression with corticosteroids is likely to be at best ineffective and at worst harmful based on a retrospective cohort study of 31 patients with PAP who received corticosteroids for at least 1 month [144]. Of these patients, 74% deteriorated during treatment with corticosteroids including emergence of new infections in some cases, highlighting the therapeutic challenges in the management of coexistent autoimmunity and immunodeficiency.

The published evidence for clinical efficacy of plasmapheresis in patients with anti-GM-CSF and anti-IFNγ autoantibodies is limited to case reports, with variable responses described. One patient showed improvement in lung disease and reduction in anti-GM-CSF titers with plasmapheresis [145]; however, a subsequent patient did not show clinical improvement despite a falling antibody titer [146]. Plasmapheresis, in conjunction with cyclophosphamide, was reported to reduce antibody titer in association with partial clinical response in one patient with NTM infection and anti-IFNγ autoantibodies [38]. Plasmapheresis does not selectively remove the AAb in question, and clinical improvement may be consequence of removal of other deleterious biomolecules, which may account for the variability in clinical outcomes in these patients.

There is promising evidence emerging for the efficacy of rituximab, an anti-CD20 monoclonal antibody that leads to depletion of B lymphocytes, in anticytokine AAb-mediated immunodeficiency. An open label trial of rituximab in 10 patients with PAP reported improvement in seven cases [147], while case reports of patients with anti-IFNγ autoantibodies and treatment-refractory NTM infection have described the efficacy of rituximab in inducing and maintaining disease remission [16,148]. While only a limited number of cases have been described, no infective complications or other severe adverse events following rituximab have been reported [16,147,148].

Antibody-mediated PRCA has been successfully treated with immunosuppression, including corticosteroids, cyclosporine A, cyclophosphamide, mycophenolate mofetil, and rituximab. However, kidney transplant is the definitive treatment for PRCA, likely due to the high dose regimen of immunosuppressants [129,130,131,149].

## 6. Conclusions

The contribution of anti-cytokine AAbs to immune dysregulation, autoimmunity and immune deficiency continues to be a fascinating area of research. These antibodies, in healthy individuals, may contribute to maintaining homeostasis of the immune response. However, in combination with genetic and environmental perturbations, anti-cytokine AAbs have the potential to severely disrupt immune processes with serious consequences in some cases. It is necessary to consider evaluation of patients for the presence of anti-cytokine AAbs in cases where disruption of normal immune processes appears to contribute to disease.
